# Machine Learning to Identify Point-of-Care Ultrasound and Evaluate Standardized Documentation: Retrospective Operational Cohort Study

**DOI:** 10.2196/84454

**Published:** 2026-08-07

**Authors:** Kevin Nguyen, Zewen Wu, Chu-An Tsai, John Vandervest, D’Anna Lammers, Ruth Cassidy, Zachary Murphy, Balaji Pandian, Maya M Hammoud, Jennifer Collin, Roger Smith, Rosalyn Maben-Feaster, Amy Kaufman Eddy, Michael L Burns

**Affiliations:** 1 Department of Anesthesiology, University of Michigan, 1500 E. Medical Center Drive, Ann Arbor, MI, 48109, United States, 1 734-936-4280; 2 Department of Obstetrics and Gynecology, University of Michigan, Ann Arbor, MI, United States; 3 Department of Anesthesiology, Weill Cornell Medicine, New York, NY, United States

**Keywords:** point-of-care ultrasound, electronic health records, medical informatics, obstetric labor, workflow, revenue cycle, machine learning

## Abstract

**Background:**

Point-of-care ultrasound (POCUS) is integral to obstetrics and gynecology (OBGYN), offering bedside diagnostic and therapeutic advantages. Despite its widespread adoption, accurate documentation and billing remain challenging due to inconsistent workflows, variable free-text note quality, and inefficiencies within electronic health record (EHR) systems. These barriers often result in missed procedural charges and hinder operational, educational, and reimbursement efforts.

**Objective:**

This study leveraged machine learning (ML) to automatically identify POCUS procedures within clinical notes and assessed the effect of implementing standardized procedure documentation (ProcDoc) templates on billing capture accuracy and efficiency.

**Methods:**

We conducted a multipart retrospective cohort study at a large academic medical center using EHRs from January 2018 to August 2024 across 11 OBGYN clinic sites. ML models (LightGBM [light gradient boosting machine] and BioClinBERT [biomedical and clinical bidirectional encoder representations from transformers]) were trained on clinical encounter notes to classify POCUS procedures and validated against Current Procedural Terminology (CPT) manual code assignments. In February 2023, a standardized ProcDoc smart form was introduced to streamline POCUS documentation and automatically trigger CPT billing codes. Preintervention and postintervention periods were compared using ML metrics and manual billing audits. Outcomes included model accuracy, recall, precision, adoption rates, improvement in billing recapture, and usage of ProcDoc templates.

**Results:**

A total of 559,029 encounters from 109,776 unique patients were analyzed. The BioClinBERT model (accuracy 0.97; *F*_1_-score 0.55-0.63) demonstrated a robust ability to identify documented and missed procedures in free-text clinical notes. ProcDoc adoption reached 75.1% within 12 months, supported by comprehensive staff education. Billing recapture—the proportion of charges missed by providers but later identified—dropped from 10.0% preintervention to 2.4% postintervention, primarily arising from the shift toward auto-capturing documentation (odds ratio 0.22, 95% CI 0.17‐0.30; *P*<.001), with overall POCUS billing slightly increased (+0.6%). Most postintervention CPT codes (1812/2404, 75.4%) originated from ProcDoc templates, confirming improved workflow efficiency and reduced manual audit burden. Model analysis and billing metrics demonstrated that improvements were associated with workflow changes and not an increase in procedure frequency.

**Conclusions:**

ML modeling proved effective for extracting POCUS procedures from clinical documentation and serving as an evaluation tool for workflow interventions. Standardized documentation with ProcDoc significantly enhanced charge capture accuracy and reduced dependence on manual chart reviews and billing reconciliation. This approach highlights the use of ML as a retrospective auditing and evaluation tool for assessing clinical workflow interventions. Broader application of similar strategies could address documentation inefficiencies and promote sustainability across health care settings.

## Introduction

Point-of-care ultrasound (POCUS) procedures are limited bedside studies that aid in diagnostic assessments and guide therapeutic interventions. These tests are increasingly used in medicine, integrated into training, and widely adopted in obstetrics and gynecology (OBGYN). They differ from traditional ultrasound by addressing a specific clinical question and providing multiple rapid and reliable benefits. POCUS is cost-effective, displays images in real time, and is operated by clinical providers who can correlate the findings with symptoms and provide immediate targeted treatments [1]. Proper capture of these procedures requires classification into Current Procedural Terminology (CPT) codes, which are used in research, training, quality improvement, and reimbursement efforts [2]. POCUS procedures are susceptible to electronic health record (EHR) capture inefficiencies due to operational variability in training, equipment, and workflows. Appropriate documentation and subsequent charge capture require provider mindfulness and manual review and capture of clinical documentation by billing teams. This is especially true in settings with frequent and widespread use, such as OBGYN visits, leading to inconsistent documentation and uncaptured performed procedures [3]. AI applications, such as machine learning (ML), offer an opportunity to identify procedures from clinical documentation. Additionally, simplifying the documentation and billing workflows for these bedside procedures offers the potential for improved capture.

In OBGYN, proper documentation and reporting of POCUS procedures are critical to reimbursements, research, diagnostic integrity, operations, and legal record-keeping [2]. Common reasons leading to inaccuracies include a lack of formal provider education, inadequate clinical documentation, and the absence of quality assurance feedback systems aimed at correcting billing errors [3]. In the emergency department, another setting in which POCUS is frequently used, quality improvement interventions have been associated with increased capture rates, reducing unbilled and nonarchived POCUS examinations [4-8]. One study found that only a small minority of emergency medicine practitioners received reimbursement for POCUS from Medicare beneficiaries and hypothesized that most POCUS examinations performed were not billed [8].

POCUS use is documented in the EHR through various methods, including standardized procedure notes, templates, or, more commonly, through free-text documentation in patient clinic visit notes, as is done at our institution. Free-text clinical documentation is variable in quality and contains documentation error rates as high as 10%, increasing the complexity of identifying the use of POCUS from this type of documentation [9]. Manual chart review of clinic notes is an arduous task, given the vast amounts of EHR documents generated daily. Advances in natural language processing, a subset of AI, allow automated insights into EHR data, including note classification, entity recognition, and text summarization [10]. Identifying examinations from EHR notes falls into an ML task known as classification [11], which has previously been successful in health care applications, such as identifying aortic stenosis [12], type 2 diabetes [13], billing assignments [14,15], and predicting miscarriages and stillbirths [16,17]. Based on the transformer architecture that underlies large language models, BioBERT (biomedical bidirectional encoder representations from transformers) is a pretrained biomedical language representation model specifically adapted for biomedical text. BioBERT is an extension of the general-purpose language model BERT (bidirectional encoder representations from transformers) and has shown superior performance on biomedical language tasks [18]. For certain tasks, studies have shown that a fine-tuned BioBERT model outperforms foundational large language models and logistic regression methods [19]. In this multipart retrospective study, we hypothesized that (1) ML modeling could predict OBGYN POCUS procedures from clinical notes, and (2) this modeling could be used to evaluate workflow changes for standardized procedural documentation.

## Methods

### Study Design and Setting

This is a multipart retrospective cohort study to (1) develop an ML model for identifying POCUS procedures from clinical notes and (2) use the ML model to evaluate the effectiveness of introducing standardized procedure documentation (ProcDoc) for POCUS procedures. This study used records from 11 unique OBGYN clinic sites within the University of Michigan-Health system, all using the identical EHR system. Four distinct datasets were used in this study: (1) The “Development (Dev)” dataset was created from all OBGYN visits from January 1, 2018, to December 31, 2020. This dataset alone was used to create the ML model in this study. (2) The first inference set, “preintervention,” included visits from February 1, 2022, to December 31, 2022. The intervention, described in detail below, occurred in February 2023. (3) The “post(1)-intervention” dataset included visits from February 1, 2023, to December 31, 2023. (4) The final dataset, “post(2)-intervention,” included visits from January 1, 2024, to August 31, 2024. The preintervention and post(1)-intervention datasets were aligned with each other and the February intervention, resulting in February to December comparisons and a 1-month gap (January 2023) in the data timeline. There was an approximately 13-month gap between January 2021 and January 2022, during which the OBGYN teams determined next steps to improve POCUS billing capture. Clinic sites slightly differed between the Dev dataset and all other datasets. The Dev dataset consisted of 10 unique clinical sites (Table S1 in Multimedia Appendix 1), whereas the preintervention, post(1)-intervention, and post(2)-intervention datasets contained data from 9 clinic sites, 8 of which overlapped with the Dev dataset. One clinic was added, and 2 clinics were removed from the ProcDoc intervention as they had significantly different workflows and EHR capture methods that were incompatible with the ProcDoc intervention. Prior to dataset creation, 2 reviewers (DL and MB) manually identified 4 important clinical free-text note types (model features) used to document POCUS procedures: progress, addendum, procedure, and admission history and physical notes. Datasets were created using all data from these predefined note types during each dataset period, with no additional inclusion or exclusion criteria applied.

### Ethical Considerations

This study was approved by the University of Michigan Institutional Review Board with a waiver of informed consent (HUM00203986), and the authors followed the STROBE (Strengthening the Reporting of Observational Studies in Epidemiology) guidelines [20] (Checklist 1). Data were deidentified; however, cases selected for manual review were reidentified solely for this purpose.

### Data Extraction and Text Preprocessing

To prepare data for ML modeling, EHR data were collected for each clinic visit encounter and preprocessed as follows. For each encounter, all notes of these types were concatenated and preprocessed by lowercasing, whitespace removal, text tokenization (using the NLTK library [21]), text stemming (through the Porter stemmer), and stripping of common words. These steps were performed only for the lightweight gradient-boosting machine (LightGBM) model. BioClinBERT (biomedical and clinical bidirectional encoder representations from transformers) model did not undergo any of these steps. Instead, since BioClinBERT has a sequence length limit of 512 tokens, keyword-based extractive summarization was applied to each document. This step retained only sentences containing at least 1 of 100 specified keywords identified using LightGBM feature importance, which ranks the most influential keywords driving the classification predictions of the LightGBM model (Table S2 in Multimedia Appendix 1) [22]. Keywords were initially statistically selected and pruned through manual review (DL and MB) of the sentences captured based on their clinical utility, keeping the top 100 keywords and omitting terms that captured only sentences containing nonclinical artifacts. This was done on the Dev dataset alone and frozen for use across all datasets. After preprocessing, documents that exceeded the sequence length limit were truncated to the first 512 tokens. The stopwords Python library “wordcloud” was used during preprocessing to determine the top 100 words, but no stopwords were used in the final model preprocessing, thus maintaining both negation cues and clinical context.

### Model Development and Model Evaluation

Before the intervention of standardized procedural documentation, POCUS procedures were manually captured from free-text clinic visit notes. To understand this manual capture process, 3 algorithms (2 types of ML models and 1 single-word search method) were developed to classify POCUS procedures from clinical documentation. The word search method simply searched for the term “ultrasound” in the processed encounter text and was used as a baseline comparison to a simplistic method.

First, a gradient boosting tree–based ML model, LightGBM, was used. Second, we used keyword insights from LightGBM to develop a BioClinBERT model, which builds on BioBERT and has been pretrained using all clinical notes from MIMIC-III (Medical Information Mart for Intensive Care III). Both models used a stratified split based on calendar years, using 80% of the data for training and reserving 20% for hold-out testing. Five-fold cross-validation of the 80% training dataset was used to tune model hyperparameters. During tuning, model weights were initialized using BioBERT-Base (version 1.0), along with PubMed 200K and PMC 270K, and the official tokenizer was adapted specifically for clinical text. Training splits for model development were grouped using encounter ID, independent of patient ID. The maximum input sequence length (including padding) was set to 512 tokens, and optimization was performed using the AdamW optimizer. The selected hyperparameters included a batch size of 16 and a learning rate of 2×10^5^. Final models were retrained using the full development dataset with the selected hyperparameter configuration. The classification threshold was set at the default value of 0.5.

BioClinBERT was subsequently evaluated on the preintervention and post(1)-intervention datasets. Standard metrics were assessed, including accuracy, recall, precision, and *F*_1_-score. The gold-standard reference label for whether a POCUS procedure was performed during an encounter was determined by the presence of one or more of 12 POCUS-specific CPT codes: 58340, 76801, 76802, 76815‐76819, 76830, 76831, 76856, and 76857 (Table S3 in Multimedia Appendix 1). Manual validation was conducted by a certified clinical documentation specialist (DL) on a random sample of encounters in which the model predicted a procedure, but no corresponding POCUS CPT codes were billed (“false positives”) and on another random sample of encounters in which the model failed to identify a billed POCUS procedure (“false negatives”).

### ProcDoc Intervention

From the preintervention evaluation, the OBGYN group identified 9 clinics for workflow interventions, 8 of which were included in the Dev dataset (Table S1 in Multimedia Appendix 1). The OBGYN team designed and implemented standardized POCUS procedure documentation (ProcDoc) templates (Figure S1 in Multimedia Appendix 1). The intervention occurred in February 2023 using Epic Smart Forms (Figure S1 in Multimedia Appendix 1), which provide customizable fields and selections for capturing indications, impressions, and other relevant details. The ProcDoc was designed to be used with OBGYN POCUS procedures and, once completed, was organized under the Procedures tab in the EHR. Submission of the ProcDoc automatically triggered the associated CPT billing code. Staff education coincided with the rollout and included formal presentations, video demonstrations, and ad hoc peer-to-peer teaching. Education was disseminated during staff meetings to faculty, residents, advanced practice providers, and certified nurse midwives, and was included in onboarding for new hires and new resident cohorts. Metrics on individual provider usage were collected, and reminders were sent to providers who displayed low rates of ProcDocs usage. The ProcDoc template was not mandatory to close an encounter. Reports on provider usage of the ProcDoc were compiled periodically. If a provider was identified as not using the ProcDoc, they were sent a tipsheet and reminder notifying them that ProcDoc was the preferred method for documentation and charge capture of clinic-performed POCUS examinations.

### ProcDoc Intervention Evaluation and Statistical Analyses

There were 2 main evaluations: ML-based and billing team–based evaluations. ML-based evaluations used the Dev, preintervention, and post(1)-intervention datasets, whereas billing team–based evaluations used the preintervention, post(1)-intervention, and post(2)-intervention datasets. For ML-based evaluations, the BioClinBERT ML model was used to label encounters. BioClinBERT was not applied to the post(2)-intervention dataset, as this dataset existed in the OBGYN billing evaluations and was inconsistent with the modeling time periods for this study. Following initial training on the Dev dataset, this model remained fixed throughout the evaluations of the preintervention and post(1)-intervention datasets.

For billing team–based evaluations, the OBGYN billing team tracked the following 3 key metrics: (1) total outpatient ultrasound billing—charges for the 2 most common OBGYN POCUS CPT codes (76815 and 76857), (2) ultrasound billing recaptures—charges that were originally missed by providers but later recaptured by the billing reconciliation and audit teams, and (3) ProcDoc adoption rates—the proportion of ultrasound charges derived from ProcDocs out of all charges. Review recapture was used as an objective outcome to estimate efficiency improvement from the ProcDoc intervention conducted by the OBGYN billing team and was determined separately from ML results and evaluations. The methodology used by the billing team involved a manual review consisting of reading each note to find missing POCUS charges. They reviewed every note affiliated with each encounter to determine appropriate charges to capture. The intensity, staffing, specific ultrasound procedural focus, reconciliation processes, and audit workflows of the billing team’s review process remained stable throughout the study.

Billing team metrics were assessed on the preintervention, post(1)-intervention, and post(2)-intervention data. To compare distributional summaries of covariates between datasets, we calculated pairwise standardized differences. An absolute standardized difference threshold exceeding 0.2 was used to indicate imbalance. We calculated the rates and odds of recapture for the preintervention, post(1)-intervention, and post(2)-intervention time periods. We then calculated odds ratios, along with corresponding 95% CIs and *P* values, for both postintervention time periods, using the preintervention period as the reference. For ease of interpretability, we also reported the inverse of the odds ratio estimates. Rates and CIs were adjusted to account for repeated measures by patient through a mixed-effects logistic model with random intercepts corresponding to the unique patient identifier and a compound symmetry covariance structure.

## Results

### Dataset Characteristics

**Figure 1.**
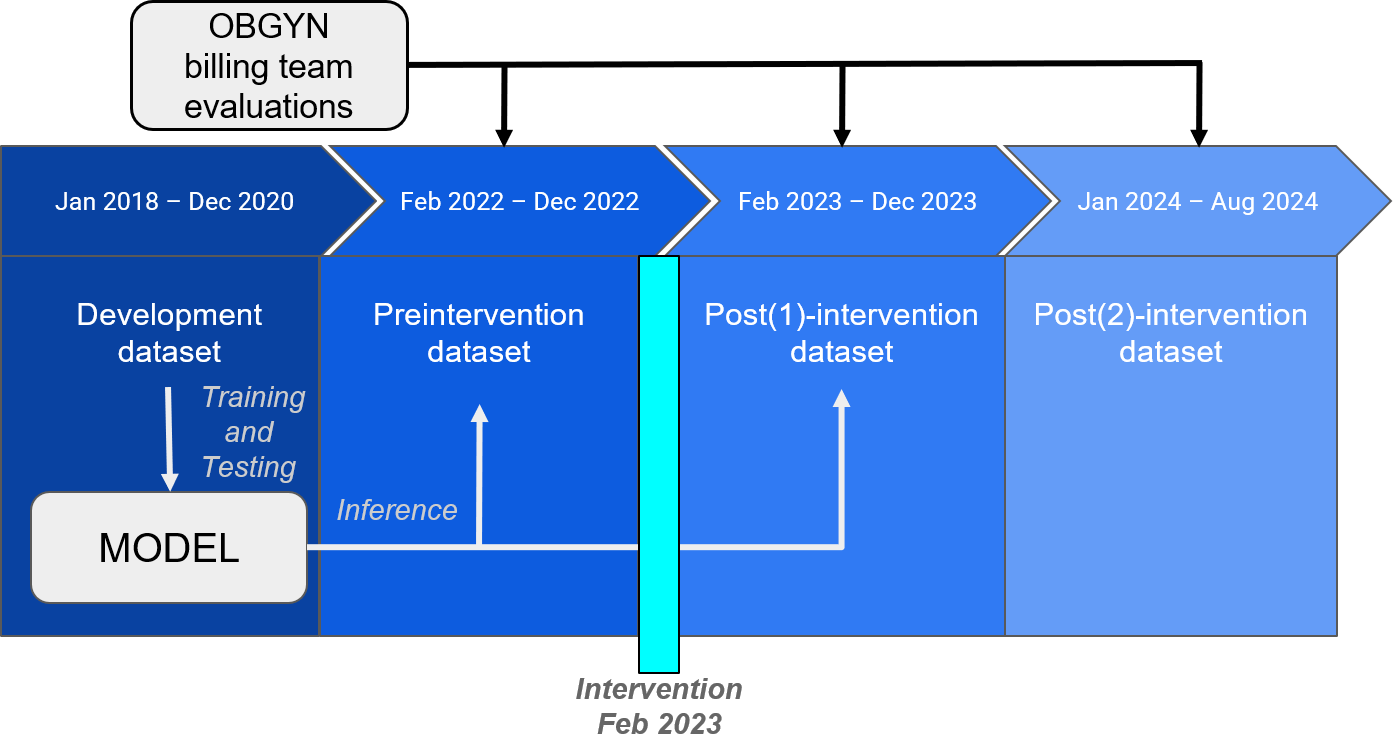
Study flowchart for AI model development, inference, and obstetrics and gynecology (OBGYN) billing team evaluations. A single AI model was developed and used to evaluate the preintervention and post(1)-intervention data. The intervention included standardized documentation (ProcDoc) implementation and clinical workflow education. OBGYN billing team evaluations spanned preintervention, post(1)-intervention, and post(2)-intervention datasets.

A total of 559,029 clinical encounters from 109,776 unique patients were included across the 4 datasets: Dev, preintervention, post(1)-intervention, and post(2)-intervention (Figure 1; Table 1). The average patient age ranged from 36.0 (SD 12.2) to 38.5 (SD 14.1) years, with no significant differences between datasets. Between 10.6% (33,643/316,390) and 11.5% (10,978/95,696) encounters were identified as Black patients, and between 4.3% (13,714/316,390) and 5.4% (4980/91,627) were identified as Hispanic. The POCUS rate identified by CPT codes was 9.9% (31,336/316,390 encounters) in the Dev dataset and ranged from 3.6% (3414/95,696) to 4.3% (2402/55,316) in the other datasets, a difference attributed to clinic inclusion and exclusion after model development (Table S1 in Multimedia Appendix 1). ProcDoc implementation focused on primary OBGYN clinics, which omitted 2 clinics included in the Dev dataset and added a clinic that was not included in the Dev dataset. All absolute standardized differences between datasets were ≤0.2.

**Table 1.** Dataset characteristics^a^.

Characteristics	Development, Jan 2018 to Dec 2020	Preintervention, Feb 2022 to Dec 2022	Post(1)-intervention, Feb 2023 to Dec 2023	Post(2)-intervention, Jan 2024 to Aug 2024	Standardized difference			
					Pre to Dev	Post(1) to Dev	Post(1) to Pre	Post(2) to Post(1)
Patients, n^b^	68,809	40,132	40,429	30,963	—^c^	—	—	—
Encounters, n	316,390	95,696	91,627	55,316	—	—	—	—
POCUS rate, encounter n (%)^d^	31,336 (9.9)	3414 (3.6)	3358 (3.7)	2402 (4.3)	—	—	—	—
Note types per encounter, mean (SD)	1.2 (0.4)	1.1 (0.3)	1.1 (0.4)	1.2 (0.4)	−0.17	−0.08	0.09	0.2
Age (y) mean (SD); missing n (%)	36.0 (12.2); 18 (0)	36.7 (13.2); 27 (0)	37.5 (13.5); 33 (0)	38.5 (14.1); 0 (0)	0.06	0.12	0.06	0.08
Weight (lbs), mean (SD); missing n (%)^e^	173.2 (45.5); 64,913 (20.5)	175.0 (45.4); 19,275 (20.1)	176.5 (46.2); 17,824 (19.5)	176.1 (46.1); 12,278 (22.2)	0.04	0.07	0.03	−0.01
Height (inches), mean (SD); missing n (%)^e^	64.5 (3.0); 176,892 (55.9)	64.5 (2.9); 60,211 (62.9)	64.5 (3.0); 60,723 (66.3)	64.4 (3.1); 38,409 (69.4)	0.01	−0.01	−0.01	−0.02
BMI, median (IQR); missing n (%)^e^	27.8 (24.0-32.9); 64,669 (20.4)	28.2 (24.2-33.5); 20,542 (21.5)	28.4 (24.4-33.7); 19,763 (21.6)	28.4 (24.3-33.7); 13,868 (25.1)	0.05	0.08	0.03	−0.004
Race, encounter n (%)					0.05	0.07	0.04	0.02
White	240,663 (76.1)	72,335 (75.6)	68,919 (75.2)	41,647 (75.3)				
Black	33,643 (10.6)	10,978 (11.5)	10,196 (11.1)	6012 (10.9)				
Asian	23,880 (7.5)	6295 (6.6)	5885 (6.4)	3615 (6.5)				
American Indian	14,285 (4.5)	4749 (5.0)	5114 (5.6)	3031 (5.5)				
Unknown, missing, or refused	3919 (1.2)	1339 (1.4)	1513 (1.7)	1011 (1.8)				
Ethnicity, encounter n (%)					0.03	0.05	0.02	0.03
Hispanic	13,714 (4.3)	4761 (5.0)	4980 (5.4)	2879 (5.2)				
Non-Hispanic	295,702 (93.5)	88,779 (92.8)	84,455 (92.2)	50,889 (92.0)				
Unknown, missing, refused	6974 (2.2)	2156 (2.3)	2192 (2.4)	1548 (2.8)				
Payer, encounter n (%)					0.12	0.15	0.03	0.04
BCBS^f^	222,034 (70.2)	64,264 (67.2)	59,909 (65.4)	36,076 (65.2)				
Commercial	70,056 (22.1)	26,005 (27.2)	25,962 (28.3)	15,868 (28.7)				
Military	1439 (0.5)	478 (0.5)	406 (0.4)	157 (0.3)				
Medicaid	4827 (1.5)	893 (0.9)	914 (1.0)	639 (1.2)				
Medicare	12,023 (3.8)	3446 (3.6)	3436 (3.7)	1840 (3.3)				
Worker’s comp	5 (0.0)	0 (0.0)	3 (0.0)	3 (0.0)				
Unknown or Missing	6006 (1.9%)	610 (0.6%)	997 (1.1%)	733 (1.3%)				

a Dataset demographics: Dev (development), Pre (preintervention), and Post(1) and Post(2) (2 postinterventions) datasets. Race categories: White (White or Caucasian only), Black (Black or African American), Asian (Asian or Pacific Islander, not Black, not Native), and American Indian (American Indian and Alaska Native or Middle Eastern/North African or Other). The Dev point-of-care ultrasound (POCUS) rate examined the rate of ultrasounds performed in the clinics within the Dev dataset, whereas the pre-post POCUS rate analyzed clinics from the preintervention and postintervention datasets and extended to all other datasets. Demographic variables were neither incorporated into the machine learning pipeline nor used in billing metrics.

b A total of 109,776 unique patients are represented across all datasets.

c Not applicable.

d POCUS rate variation is based on variations in clinic inclusion (Table S1 in Multimedia Appendix 1).

e BMI was recorded independent of height and weight in these patient populations.

f BCBS: Blue Cross Blue Shield.

### Model Development and Preintervention Evaluation

The same ML model was applied without retraining at any stage of the project to retain focus and consistency throughout preintervention and postintervention comparisons. Using the Dev dataset, 3 approaches were evaluated to predict OBGYN POCUS procedures from free-text in clinical notes: word search, LightGBM, and BioClinBERT. The intention of using a word search was to compare a simple, unoptimized search strategy. Word search yielded accuracy, recall, precision, and *F*_1_-score of 0.86, 0.59, 0.36, and 0.44, respectively, whereas LightGBM (0.95, 0.90, 0.65, and 0.76, respectively) and BioClinBERT (0.97, 0.79, 0.86, and 0.82, respectively) performed better. Results analyzed across distinct OBGYN locations showed an average site accuracy of 0.93 (SD 0.11), with a maximum of 0.98 and a minimum of 0.67. Only 2 of 10 sites showed a model accuracy of less than 95%, each containing the lowest number of patient encounters; A total of 9.9% (n=31,336) of the Dev dataset encounters contained one or more of the associated CPT codes (Table 1; Table S1 in Multimedia Appendix 1). Model results varied by clinic (Table S4 in Multimedia Appendix 1). BioClinBERT ML modeling labeled an additional 1.1% of encounters as positive, representing an 11.6% increase compared to CPT codes. The gold-standard label for model training used existing billing codes, which contained missed billing encounters and thus are variable and potentially unreliable. While it is infeasible to manually review notes from each encounter, validation was conducted (by reviewer DL) on simple random samples from the Dev dataset for false positives and false negatives. False positive samples consisted of 101 encounters, where the model predicted a procedure, but no associated POCUS CPT codes were billed. Of these 101 encounters, 85 (84.2%) encounters (adjusted 84.0% with 95% CI 76.8%-91.2%) were confirmed as correct upon manual review, indicating that BioClinBERT successfully identified missed POCUS procedures not captured in billing data. A random sample of 114 false negative cases was reviewed, and 63 (55.3%; adjusted 54.9%, 95% CI 45.7%-64.1%) were found to be truly negative for a POCUS procedure.

### ProcDoc Intervention and Evaluation

**Figure 2.**
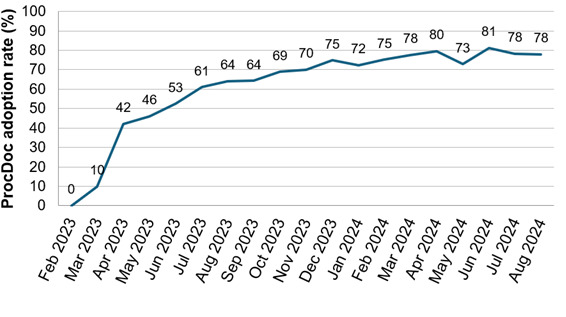
Procedure document (ProcDoc) adoption rate following the February 2023 implementation workflow intervention.

Adoption of ProcDoc rose rapidly after implementation, reaching an adoption rate of 64.2% in August 2023, 75.1% in February 2024, and 77.9% in August 2024 (Figure 2). Relative to preintervention, post(1) data were associated with improved true positive labeling, as evidenced by increased precision (0.562 vs 0.487), recall (0.711 vs 0.635), and positive likelihood ratio (36.2 vs 28.2), indicating improved true positive labeling after the intervention (Table 2). There was sequential improvement over time following the intervention (Figures S1-S6 in Multimedia Appendix 1). False positives (model prediction positive but no CPT codes billed) were considered a flag for possible missed billing opportunities, identifying cases that could potentially be examined further to identify missed billing charges. The false discovery rate was calculated as the percentage of cases where no CPT codes were billed out of those predicted positive in the model. However, the nature of false positives could shift over time or across different clinics, making this an imperfect measure. Post(1) encounters displayed a decreased false discovery rate (0.438 vs 0.513). Trending by month again displayed appreciable and sustained improvement postintervention (Figure S2 in Multimedia Appendix 1). The OBGYN billing team evaluated the 2 most common POCUS CPT codes: 76815 and 76857 (Table 3). These 2 codes represented more than 95% of all encounters from the training dataset. The total ultrasounds billed slightly increased from 3176 to 3195 over the same time period, preintervention to post(1)-intervention. CPT code assignment occurred during 2 distinct phases: (1) “Original,” where codes were assigned directly by providers based on initial documentation, and (2) “Review,” where codes were added later through manual review by billing reconciliation and audit teams. From preintervention to post(1)-intervention to post(2)-intervention, the percentage of review recapture decreased from 10.0% (317/3176) to 2.4% (58/2404), while capture increased, primarily due to the shift toward auto-capturing documentation, with 75.4% (1812/2404) of CPT code captures originating from ProcDocs (Table 3). The odds of review recapture for the post(2)-intervention group were 0.22 (95% CI 0.17-0.30; *P*<.001) compared to the preintervention group.

**Table 2.** Machine learning (ML) model statistical output measurements comparing preintervention and postintervention data by encounter^a^.

Dataset	ML model predicted positive		ML model predicted negative		Accuracy	*F*_1_-score	Precision rate (positive predictive value) = TP^b^ / predicted positive	Recall (sensitivity) = TP / positive	Specificity (true negative rate) = TN^c^ / negative	Positive likelihood ratio = recall / FP^d^ rate	False discovery rate = FP / predicted positive
	Billing Data POCUS CPT Yes (TP^a^)	Billing Data POCUS CPT No (FP^b^)	Billing Data POCUS CPT Yes (FN^e^)	Billing Data POCUS CPT No (TN^d^)							
Preintervention	1979	2084	1137	90,424	0.966	0.551	0.487	0.635	0.977	28.2	0.513
Post(1)-intervention	2199	1717	894	85,776	0.971	0.627	0.562	0.711	0.980	36.2	0.438

a Predicted positive is TP+FP, negative is FP+TN, positive is FN+TP. Positive was determined by the presence of one or more of 12 POCUS-specific current procedural terminology (CPT) codes: 58340, 76801, 76802, 76815-76819, 76830, 76831, 76856, and 76857 (Table S3 in Multimedia Appendix 1).

b TP: true positive.

c TN: true negative.

d FP: false positive.

e FN: false negative.

**Table 3.** Billing team evaluations by encounter over 3 windows of time: preintervention, post(1)-intervention, and post(2)-intervention^a^.

OBGYN^c^ billing team evaluations	Preintervention, Jan 2022 to Dec 2022	Post(1)-intervention, Jan 2023 to Dec 2023	Post(2)-intervention, January 1, 2024, to August 31, 2024
CPT^b^ code: 76815			
Original			
non-ProcDoc, n	2803	1359	519
ProcDoc, n	0	1570	1795
Review, n	317	171	57
CPT code: 76857			
Original			
non-ProcDoc, n	56	63	15
ProcDoc, n	0	31	17
Review, n	0	1	1
Total capture (original+review), n	3176	3195	2404
Review recapture, n (%)	317 (10.0)	172 (5.4)	58 (2.4)
Odds of review recapture	0.111	0.057	0.025
Odds ratio of review recapture (ref: preintervention)	—^d^	0.513	0.224
95% CI (ref: preintervention)	—	0.422-0.623	0.168-0.298
*P* value (ref: preintervention)	—	<.001	<.001
Inverse odds ratio of review recapture (ref: preintervention)	—	1.949	4.464
ProcDoc capture, n (%)	0 (0)	1601 (50.1)	1812 (75.4)

a Manual internal billing team evaluations for capture consisted of original evaluations and reviews. Point-of-care ultrasound (POCUS) use determines notes using the standardized documentation (ProcDoc) in the process. Adjusted estimates were calculated to account for clustering within patients. Using the subsample (8775 encounters corresponding to 7032 patients) used to calculate recapture odds, odds ratios, 95% CI, and *P* values, we fit a mixed-effects logistic model with random intercepts corresponding to unique patient identifiers using a compound symmetry covariance structure. The variance parameter estimate corresponding to the random effect of the mixed-effects logistic model was 0.068, with an SE of 0.02. Review was conducted for the 2 most common obstetrics and gynecology POCUS CPT codes (76815 and 76857; Table S3 in Multimedia Appendix 1).

b CPT: Current Procedural Terminology.

c OBGYN: obstetrics and gynecology.

d Reference, so not applicable.

## Discussion

### Principal Findings

In this study, we first developed an ML model based on the BERT architecture to identify POCUS procedures in OBGYN clinical documentation and demonstrated its effectiveness across multiple clinics within a single, large academic medical center. Second, we used this ML model to help evaluate a workflow intervention incorporating standardized documentation, with results associated with significantly improved capture of POCUS procedures. Improved capture, evidenced by both ML model performance and billing team evaluation metrics, reduced the existing manual efforts required from billing teams dedicated to this area.

This study demonstrated the use of AI to guide and evaluate clinical workflow improvements. This finite use of AI technology is unique in that health care applications of AI are primarily continuous and require EHR integration, monitoring, and maintenance solutions. This work exemplifies how AI-driven clinical workflow analyses can address systemic inefficiencies without requiring direct AI model integration and the associated costs. The transition to ProcDocs significantly improved the capture rates of POCUS procedures. This intervention not only streamlined documentation but also ensured that relevant CPT codes were automatically mapped and triggered, reducing the administrative burden, decreasing the need for recapture, and minimizing the chances of missed billing opportunities. This reduction in recapture reflects a shift in documentation volume toward auto-capturing templates that reduced the absolute manual audit burden. The minimal increase in POCUS use from preintervention to postintervention suggests that the workflow standardization was a major contributor to improved capture. The rapid adoption of the ProcDoc by clinical providers, supported by comprehensive staff education and continuous monitoring, indicates the practicality of this type of intervention.

ML modeling was not necessary to identify the challenges with billing capture, but it aided the process. Subsequently, the modeling we developed was useful for evaluating the workflow intervention and served as a backup in the event that the workflow intervention was unsuccessful. Our modeling results were strong relative to previous studies using AI in health care billing prediction. Specifically, AI to identify *ICD* (*International Classification of Diseases*) codes has been associated with low agreement with human coders (~15%) [23], while identification of anesthesiology CPT codes yielded accuracies as high as ~88% [16]. The BioClinBERT model developed in this study met or surpassed these previously reported performance metrics. These modeling results underscore the ability to classify complex medical narratives accurately, highlighting the potential of AI to enhance clinical documentation and classification. Manual chart reviews, although time-intensive and impractical due to the sheer volume of clinical notes, can be augmented or replaced by scalable and efficient AI models. The use of AI in this study was as a finite evaluation tool, where observed changes in capture were attributed to the ProcDoc intervention rather than AI modeling. This method of AI is low-cost and yielded great results. Alternatively, when using AI tools in continuous operations, costs to develop and maintain ML models can outweigh potential modest increases in captured billing [24]. One study estimated US $217,000 to develop and US $6000 to maintain each ML model. At approximately US $100 per POCUS study, this would require the capture of 60 additional POCUS exams for maintenance and 2170 for model development costs. While precise institutional costs were not formally tracked, model development required dedicated data engineering effort and secure computing infrastructure typical of academic ML workflows. While this work focuses on capturing missed revenue opportunities through workflow changes, in general, the use of AI tools could identify baseline over-billing in the human reference standard and thereby help to mitigate institutional audit risk. Specifically, the manual audit indicated a substantial error rate in the billing reference standard; it simultaneously confirmed that the BioClinBERT model failed to identify an ultrasound procedure in 51 of 114 (44.7%) of the audited false negative encounters. These considerations are imperative for return-on-investment estimations when considering AI applications.

### Comparison to Prior Work

Finally, this work expands AI applications in health care, expanding beyond obstetrics, yet adding to previous predictive AI research in the area, such as predicting miscarriages and stillbirths [16,17], successful vaginal deliveries [25], vaginal birth after cesarean delivery [26], acidemia at birth [27], postpartum depression [28], and equitable considerations for the use of AI [29]. The results of this study are applicable to health care billing at large, where the quality of documentation and capture is immensely important for organizational sustainability and quality of care [30]. Reliable capture and reporting of clinical procedures are crucial for patient care, diagnostic accuracy, operational efficiency, and appropriate reimbursement—key factors in both the success and quality of health care services. As interest in AI-driven payer denial processes grows, further research is needed to integrate AI into health care revenue cycle operations, particularly in coding and billing workflows, to improve financial sustainability and streamline reimbursement [31].

### Limitations

Despite considerable improvements using this strategy, several limitations exist. (1) The study was conducted on a single procedure type, within a single health care system, using a single EHR vendor, which may limit the generalizability of findings to other clinical environments with different documentation practices and procedures. The performance of this ML model may vary across different clinics and may worsen when extrapolated to other clinics or institutions. Specifically, the ML development dataset included clinics with disproportionately high POCUS use, from which the model will likely perform better with documentation patterns specific to these settings. (2) Our ML model relied on the quality and consistency of input data, which may differ across settings due to variations in EHR maintenance, clinician documentation, billing workflows, and EHR vendor services. (3) As data changes over time due to drift and shift, ML models will be time-sensitive without retraining. (4) Our comparison of models was rigorous and iterative but not exhaustive, and there may exist improved ML models for the classification of OBGYN POCUS procedures. (5) The use of keyword extractive summarization risks reducing key components for model inputs, resulting in model underperformance. Additionally, the extractive summarization may have retained nonclinical terms and may be susceptible to shortcut associations. (6) A few clinics changed between the training and evaluation periods, causing a reduction in the POCUS+ rates in the development and subsequent datasets. This reduction could lead to misbalanced datasets that are less representative of the model training dataset, causing temporal biases in the modeling, specifically concerning overfitting training datasets and high-volume clinics. (7) There exist noncontiguous time windows and multicomponent interventions in this study, and other time-varying factors and cointerventions may contribute to the observed reductions in recapture and improved model-CPT agreement. (8) While false positives of the model may be interpreted as potential missed billing opportunities and validated with a small case review of randomly sampled cases, more exact determinations of missed billing rates would require extensive records reviews that were beyond the scope of this project and our institution’s current capacity. (9) Training splits for model development were grouped using encounter ID, independent of patient ID. As a patient may have multiple encounters within each dataset, the same patient may be represented multiple times across different datasets, though each encounter was unique. (10) As the keyword vocabulary was fixed using 2018 to 2020 data, the method may be sensitive to temporal drift in clinical language, potentially missing newer terms or language changes introduced after 2020. (11) Billing team evaluations addressed only the 2 most common CPT codes, omitting approximately 5% of codes used for this type of exam. (12) Analysis of administrative improvements is strictly conditional on an encounter eventually being billed, leaving the true baseline of total procedures performed unknown. (13) The comparison against a single-term word search may overstate the apparent performance gap relative to a more comprehensive clinical search string. (14) Finally, restricting model input to 4 note types may cap the model’s recall and inflate the apparent false negative count relative to institution-wide billing capture.

### Future Directions

Future research should aim to validate these findings across diverse health care settings and specialties to establish broader applicability. Our findings may encourage similar interventions in other medical departments where procedural documentation is critical. We advocate for continued exploration and integration of AI tools in clinical practice to enhance accuracy, efficiency, and sustainability in health care delivery. Specifically, applying ML for real-time detection of missed charge capture opportunities holds promise for further reduction of manual labor and improvement of administrative efficiency.

### Conclusions

ML modeling proved effective for extracting POCUS procedures from clinical documentation and serving as an evaluation tool for workflow interventions. Standardized documentation with ProcDoc significantly enhanced accurate charge capture and reduced dependence on manual chart reviews and billing reconciliation. This approach highlights the use of ML as a retrospective auditing and evaluation tool for assessing clinical workflow interventions. Broader application of similar strategies could address documentation inefficiencies and promote sustainability across health care settings.

## Supplementary material

10.2196/84454 Multimedia Appendix 1 Ultrasound rates by clinic, model inputs, and performance over time.

10.2196/84454 Checklist 1 STROBE checklist.

## Abbreviations

BERT: bidirectional encoder representations from transformers
BioClinBERT: biomedical and clinical bidirectional encoder representations from transformers
CPT: Current Procedural Terminology
EHR: electronic health record
*ICD*: *International Classification of Diseases*
LightGBM: light gradient-boosting machine
MIMIC-III: Medical Information Mart for Intensive Care III
ML: machine learning
OBGYN: obstetrics and gynecology
POCUS: point-of-care ultrasound
ProcDoc: procedure documentation
STROBE: Strengthening the Reporting of Observational Studies in Epidemiology
