# Supplementary material for: Machine Learning to Identify Point-of-Care Ultrasound and Evaluate Standardized Documentation: Retrospective Operational Cohort Study
Source: J Med Internet Res. 2026 Aug 7;28:e84454. doi: 10.2196/84454 (PMC13451011; doi:10.2196/84454)
Supplement: Supplementary file 1 [file jmir-v28-e84454-s001.docx]

**Supplemental Digital Content**

|  | **Page** |
| --- | --- |
| **Table S1: Ultrasound rates by clinic** | 1 |
| **Table S2: Important text keywords** | 2 |
| **Table S3: Billing codes** | 3 |
| **Table S4: Model evaluations by clinic** | 4 |
| **Figure S1: Procedure document (ProcDoc)** | 5 |
| **Figure S2: Model false discovery rate over time** | 6 |
| **Figure S3: Model recall over time** | 7 |
| **Figure S4: Model precision over time** | 8 |
| **Figure S5: Model specificity over time** | 9 |
| **Figure S6: Model positive likelihood ratio over time** | 10 |

| **Clinic** | **Dev** | **Pre** | **Post(1)** | **Post(2)** |
| --- | --- | --- | --- | --- |
| **1** | 1.4%  (1405 / 98701) | 0.6%  (197 / 30340) | 0.7%  (206 / 29537) | 1.3%  (245 / 19597) |
| **2** | 6.1%  (2618 / 42656) | 4.3%  (574 / 13247) | 4.8%  (639 / 13239) | 5.4%  (300 / 5521) |
| **3** | 4.6%  (1161 / 25074) | 4.1%  (426 / 10345) | 2.9%  (311 / 10562) | 3.7%  (235 / 6377) |
| **4** | 4.8%  (866 / 18178) | 4.1%  (269 / 6542) | 6.1%  (354 / 5780) | 8.7%  (355 / 4092) |
| **5** | 5.5%  (1809 / 32991) | 5.1%  (567 / 11186) | 5.1%  (539 / 10469) | 5.8%  (408 / 7023) |
| **6** | 4.5%  (1286 / 28855) | 6.2%  (661 / 10703) | 6.8%  (673 / 9881) | 8.5%  (445 / 5232) |
| **7** | 22.1%  (115 / 520) | 14.4%  (28 / 194) | 28.2%  (62 / 220) | 19.3%  (31 / 161) |
| **8** | 4.5%  (1445 / 32087) | 6.2%  (588 / 9443) | 6.0%  (507 / 8490) | 6.3%  (327 / 5172) |
| **9*** | 56.3%  (19787 / 35175) | Not included | Not included | Not included |
| **10*** | 39.5%  (844 / 2135) | Not included | Not included | Not included |
| **11^₶^** | Not included | 2.8%  (104 / 3669) | 2.0%  (67 / 3416) | 2.7%  (56 / 2109) |
| **TOTAL Encounters (#)** | **316,390** | **95,696** | **91,627** | **55,316** |
| **Missing or conflicting clinic label (#)** | **18** | **27** | **33** | **32** |
| **Encounters with POCUS (%)** | **9.9%** | **3.6%** | **3.7%** | **4.3%** |
| **Dev POCUS Rate (%)** | **9.9%^‡^** | 9.7% | 11.0% | 10.7% |
| **Pre-Post POCUS Rate (%)** | 3.8% | **3.6%^‡^** | **3.7%^‡^** | **4.3%^‡^** |

**Table S1: Ultrasound rates by clinic.** Percentage of clinical encounters with an OBGYN POCUS billing by unique clinic. Results are shown as % (positive OBGYN POCUS / total encounters). * = clinic unique to Development (Dev) dataset; **^₶^** = clinic unique to Pre-, Post-, and Final datasets. Dev POCUS Rate (%) is the rate of POCUS in the encounters from clinics 1-10 for the time period used in each respective dataset. Pre-Post POCUS Rate (%) is the rate of POCUS in the encounters from clinics 1-8 and 11 for the time period used in each respective dataset. ^‡^ = the POCUS rate in the actual respective dataset used in this study.

'transvaginal', 'ultrasound', 'please', 'sliup', 'bsus', 'afi', 'us', 'showed', 'needle', 'bp', 'pain', 'wd', 'menses', 'john', 'measures', 'crl', 'read', 'examinationmonitoring', 'catheter', 'labs', 'portal', 'well', 'ob', 'documented', 'uterus', 'yo', 'seen', 'routine', 'prenatal', 'objective', 'lmp', 'fibroid', 'clinic', 'take', 'pelvic', 'abd', 'intake', 'chaperone', 'achieved', 'room', 'estradiol', 'reviewed', 'pt', 'new', 'cycle', 'bedside', 'md', 'fetal', 'thyroid', 'assessment', 'note', 'present', 'liver', 'discussed', 'mm', 'sono', 'plan', 'patient', 'consent', 'call', 'transabdominal', 'cm', 'reactive', 'cavity', 'orders', 'without', 'skin', 'performed', 'vb', 'normal', 'arm', 'panorama', 'demonstrates', 'gp', 'treatment', 'ovary', 'systems', 'examination', 'visit', 'see', 'gbs', 'segment', 'presents', 'daily', 'care', 'noted', 'informed', 'hr', 'history', 'practice', 'misoprostol', 'tvcl', 'procedure', 'chief', 'rtc', 'lof', 'given', 'fm', 'vertex', 'sab'

#### **Table S2: Important text keywords.** The top one hundred keywords were selected using LightGBM’s (lightweight gradient-boosting machine) Feature Importance, which ranks the most influential keywords that drive the LightGBM model’s classification predictions. Only sentences containing one or more of these keyword features were kept in preprocessing for the BioClinBERT model. Manual review of included sentences was used to remove words that led to inclusion of clinically unimportant sentences.

| **CPT Code** | **Code Category** | **Description** |
| --- | --- | --- |
| 58340 | Under Introduction Procedures on the Corpus Uteri | Introduces a catheter with saline or contrast material into the woman's uterus or the fallopian tubes at the same time as ultrasound or X–ray imaging. This procedure is done to examine the uterus and fallopian tubes for a blockage or abnormality |
| 76801 | Under Diagnostic Ultrasound Procedures of the Pelvis Obstetrical | Ultrasound of a pregnant uterus for examination of the first or a single fetus and mother during the first trimester, or less than 14 weeks of pregnancy, using a transabdominal approach |
| 76802 | Under Diagnostic Ultrasound Procedures of the Pelvis Obstetrical | Transabdominal ultrasound to examine each additional gestational sac during the first trimester |
| **76815*** | Under Diagnostic Ultrasound Procedures of the Pelvis Obstetrical | Limited examination of the fetus using ultrasound |
| 76816 | Under Diagnostic Ultrasound Procedures of the Pelvis Obstetrical | Reexamines the fetus to follow up on a prior abnormal transabdominal ultrasound |
| 76817 | Under Diagnostic Ultrasound Procedures of the Pelvis Obstetrical | Ultrasound of the pregnant uterus to examine the fetus and the mother's organs using a transvaginal approach |
| 76818 | Under Diagnostic Ultrasound Procedures of the Pelvis Obstetrical | Biophysical profile test to measure the health of the baby during pregnancy. The test consists of a nonstress test and an ultrasound |
| 76819 | Under Diagnostic Ultrasound Procedures of the Pelvis Obstetrical | Biophysical profile test to measure the health of the baby during pregnancy using only ultrasound and not a nonstress test |
| 76830 | Under Diagnostic Ultrasound Procedures of the Pelvis Non-Obstetrical | Transvaginal ultrasound to assess the reproductive organs, that is, the uterus, fallopian tubes, ovaries, cervix, and vagina in a female patient |
| 76831 | Under Diagnostic Ultrasound Procedures of the Pelvis Non-Obstetrical | Ultrasound examination during which he infuses saline into the uterus and may also use color flow Doppler to enhance imaging |
| 76856 | Under Diagnostic Ultrasound Procedures of the Pelvis Non-Obstetrical | Pelvic ultrasound used to assess body parts such as the bladder, ovaries, uterus, cervix, and fallopian tubes in females and the bladder, prostate gland, and seminal vesicles in males |
| **76857*** | Under Diagnostic Ultrasound Procedures of the Pelvis Non-Obstetrical | Pelvic ultrasound used to assess one or more pelvic structures, such as bladder, ovaries, uterus, cervix, and fallopian tubes in females and the bladder, prostate gland, and seminal vesicles in males |

**Table S3: Billing codes.** Current Procedural Terminology (CPT) codes for OBGYN POCUS procedures used in this study. Code categories and descriptions obtained from Codify by AAPC (American Academy of Professional Coders, <https://www.aapc.com/codes/cpt-codes/76801>, accessed Aug, 2024). * = codes chosen by OBGYN billing team for evaluation.

| **Clinic** | **Dev**  **(accuracy, F1, recall, precision)** | **Pre**  **(accuracy, F1, recall, precision)** | **Post(1)**  **(accuracy, F1, recall, precision)** |
| --- | --- | --- | --- |
| **1** | 0.949, 0.558, 0.584, 0.534 | 0.962, 0.105, 0.406, 0.060 | 0.967, 0.171, 0.523, 0.102 |
| **2** | 0.964, 0.716, 0.758, 0.678 | 0.975, 0.618, 0.737, 0.532 | 0.968, 0.663, 0.681, 0.646 |
| **3** | 0.968, 0.685, 0.685, 0.686 | 0.971, 0.645, 0.704, 0.595 | 0.979, 0.650, 0.734, 0.583 |
| **4** | 0.970, 0.715, 0.766, 0.671 | 0.966, 0.646, 0.751, 0.566 | 0.970, 0.777, 0.868, 0.704 |
| **5** | 0.974, 0.764, 0.800, 0.733 | 0.977, 0.748, 0.676, 0.838 | 0.982, 0.810, 0.759, 0.869 |
| **6** | 0.967, 0.681, 0.702, 0.662 | 0.956, 0.571, 0.573, 0.570 | 0.964, 0.691, 0.638, 0.754 |
| **7** | 0.798, 0.500, 0.643, 0.409 | 0.860, 0.228, 0.190, 0.285 | 0.831, 0.412, 0.309, 0.619 |
| **8** | 0.975, 0.767, 0.760, 0.774 | 0.963, 0.652, 0.605, 0.706 | 0.758, 0.736, 0.716, 0.758 |
| **9*** | 0.964, 0.966, 0.959, 0.973 | Not included | Not included |
| **10*** | 0.644, 0.651, 0.878, 0.518 | Not included | Not included |
| **11^₶^** | Not included | 0.975, 0.618, 0.737, 0.532 | 0.983, 0.654, 0.841, 0.535 |
| **TOTAL** | **0.969, 0.823, 0.785, 0.863** | **0.966, 0.551, 0.635, 0.487** | **0.971, 0.627, 0.711, 0.562** |

**Table S4: Model evaluations by clinic.** Accuracy, F1, recall, and precision values across all clinics used in the study by dataset. * = clinic unique to Development (Dev) dataset; **^₶^** = clinic unique to Pre-, Post-, and Final datasets.

**Figure S1: Procedure document (ProcDoc).** Example of POCUS EHR procedure documentation implemented as part of the workflow intervention started February 2023.

False Discovery Rate

False Discovery Rate

|  | 2023 | 2022 |
| --- | --- | --- |
| Feb | 0.46319 | 0.438547 |
| Mar | 0.479012 | 0.429268 |
| Apr | 0.494872 | 0.461538 |
| May | 0.442211 | 0.477922 |
| Jun | 0.479893 | 0.442254 |
| Jul | 0.38674 | 0.45584 |
| Aug | 0.370056 | 0.441624 |
| Sep | 0.375375 | 0.452096 |
| Oct | 0.349099 | 0.476323 |
| Nov | 0.378016 | 0.461929 |
| Dec | 0.347305 | 0.530667 |

**Figure S2:** **Model false discovery rate over time.** False Discovery Rate (False Positive / Predicted Positive) over time for the BioClinBERT models. False discovery rate can represent potential missed charges with the lower the value representing lower the potential missed charges. Values are plotted by month comparing Feb-Dec 2022 and Feb-Dec 2023, with intervention beginning February 2023.

**Figure S3:** **Model recall over time.** Recall (“True Positive Rate” or “Sensitivity”, True Positive / All Positives, 1 – False Negative Rate) over time for the BioClinBERT models. Values are plotted by month comparing Feb-Dec 2022 and Feb-Dec 2023, with intervention beginning February 2023.

**Figure S4:** **Model precision over time.** Precision (“Positive Predictive Value”, True Positive / Predicted Positive, 1 – False Discovery Rate) over time for the BioClinBERT models. Values are plotted by month comparing Feb-Dec 2022 and Feb-Dec 2023, with intervention beginning February 2023.

**Figure S5:** **Model specificity over time.** Specificity (“True Negative Rate”, True Negative / All Negatives, 1 – False Positive Rate) over time for the BioClinBERT models. Values are plotted by month comparing Feb-Dec 2022 and Feb-Dec 2023, with intervention beginning February 2023.

**Figure S6:** **Model positive likelihood ratio over time.** Positive Likelihood Ratio (True Positive Rate / False Positive Rate) over time for the BioClinBERT models. Values are plotted by month comparing Feb-Dec 2022 and Feb-Dec 2023, with intervention beginning February 2023.
